# Muscular contraction mode differently affects autonomic control during heart rate matched exercise

**DOI:** 10.3389/fphys.2015.00156

**Published:** 2015-05-19

**Authors:** Matthias Weippert, Martin Behrens, Ray Gonschorek, Sven Bruhn, Kristin Behrens

**Affiliations:** ^1^Institute of Sport Science, University of RostockRostock, Germany; ^2^Institute of Exercise Physiology and Public HealthRostock, Germany

**Keywords:** circulation, heart rate variability, autonomic nervous system, isometric exercise, afferent feedback, central command

## Abstract

The precise contributions of afferent feedback to cardiovascular and respiratory responses to exercise are still unclear. The aim of this crossover study was to assess whether and how autonomic cardiovascular and respiratory control differed in response to dynamic (DYN) and isometric contractions (ISO) at a similar, low heart rate (HR) level. Therefore, 22 healthy males (26.7 ± 3.6 yrs) performed two kinds of voluntary exercises at similar HR: ISO and DYN of the right quadriceps femoris muscle. Although HR was eqivalent (82 ± 8 bpm for DYN and ISO, respectively), rating of exertion, blood pressures, and rate pressure product were higher, whereas breathing frequency, minute ventilation, oxygen uptake and carbon dioxide output were significantly lower during ISO. Tidal volume, end-tidal partial pressures of O_2_ and CO_2_, respiratory exchange ratio and capillary blood lactate concentration were comparable between both contraction modes. Heart rate variability (HRV) indicators, SDNN, HF-Power and LF-Power, representing both vagal and sympathetic influences, were significantly higher during ISO. Sample entropy, a non-linear measure of HRV was also significantly affected by contraction mode. It can be concluded that, despite the same net effect on HR, the quality of cardiovascular control during low intensity exercise is significantly different between DYN and ISO. HRV analysis indicated a sympatho-vagal coactivation during ISO. Whether mechanoreceptor feedback alone, a change in central command, or the interaction of both mechanisms is the main contributor of the distinct autonomic responses to the different exercise modes remains to be elucidated.

## Introduction

Several mechanisms are thought to be responsible for the modulation of the circulatory and respiratory responses during voluntary muscle contractions: influences from higher brain centers, also called central command, and reflex activity primarily involving inputs from chemo-, mechano- and baroreceptor afferents (Rowell and O'Leary, 1990). Via the activation of neuronal circuits within the medulla oblongata each mechanism modulates the sympathetic and parasympathetic outflow (Goodwin et al., 1972; Iellamo et al., 1999a; Matsukawa, 2012). The influence of each mechanism on heart rate (HR), respiration and blood pressure (BP) responses to exercise depends on factors like recruited muscle mass, muscle fiber type, exercise intensity and presumably the contraction mode (Mitchell et al., 1980; Lewis et al., 1985; Leicht et al., 2008). Results of experimental studies suggest that chemical as well as mechanical stimuli significantly contribute to the cardiovascular and respiratory responses to exercise by activating type III and IV afferent fibers in the muscle and joints (Coote, 1975; Amann, 2012). Although the importance of skeletal muscle afferent feedback to the autonomic control of circulation and respiration in humans is incontrovertible, the precise contributions are still incompletely understood (Fisher, 2014). Early work that compared the cardiovascular response to static (ISO) and dynamic muscular actions (DYN) in humans, indicated a strong increase in HR and systolic arterial blood pressure (SBP), and minor changes in diastolic arterial blood pressure (DBP) during DYN, while ISO seem to induce only modest increases of HR but marked increases in SBP and in particular DBP (Tuttle and Horvath, 1957; Lindquist et al., 1973; Laird et al., 1979; Chapman and Elliott, 1988). At similar HR (about 90 bpm) isometric handgrip elicited a stronger increase of SBP and DBP compared to cycling (Lindquist et al., 1973). Isometric vs. dynamic contractions of identical small muscles, e.g. during submaximal handgrip exercise, elicited similar HR and BP responses (Louhevaara et al., 2000; Stebbins et al., 2002), while it was shown that submaximal isometric contractions of larger muscles (e.g. knee extensors/flexors) at 40% of maximum effort can induce lower HR and BP responses than isokinetic or isotonic contractions (Iellamo et al., 1997a). During moderate exercise intensity of the leg extensors Chapman and Elliott (1988) found a significant increase in HR and SBP during DYN, while DBP reached its highest values during ISO. Nevertheless, they concluded that if the same muscle groups are used, the effect of the exercise mode on cardiovascular response is more similar than frequently stated. Cottin et al. (2004), who compared heart rate variability (HRV) indices during a judo randori vs. ergometer cycling eliciting the same HR level, concluded that autonomic control during exercise rather depends on HR level than exercise type. This assumption is based on the finding of a similar spectral energy distribution within the low frequency (LF) and high frequency (HF) bands of HRV. However, due to the intense exercise in this study, with an average HR above 180 bpm, conclusions regarding the autonomic mode of HR control based on spectral analyses of HRV are strongly limited. HRV at greater HR-levels is often almost negligible and the remaining variance, especially within the HF band, is probably due to non-neural mechanisms (Casadei et al., 1995, 1996; Perini and Veicsteinas, 2003). Furthermore, as the location and size of the active muscles during cycling and judo exercises are different, the influence of contraction mode itself on HR control remains to be investigated.

Gonzalez-Camarena et al. (2000) compared DYN and ISO at moderate workload [DYN at 30% maximal oxygen uptake and ISO 30% maximal voluntary contraction force (MVC), respectively] and found HR being lower, BP and effort perception being higher, and respiratory rate being similar during ISO. HRV measures pointed to a stronger vagal cardiac modulation during ISO. However, as HRV is largely dependent on average HR, which was significantly different between DYN and ISO, the additional value of HRV analysis in this context is limited. Further, it is still unclear if the difference in autonomic cardiovascular response reported by these authors are caused by the contraction mode itself, e.g. by different inputs from mechanoreceptor afferents, or a different metabolic situation during the different kinds of exercise, because, compared to DYN, ISO might elicit a stronger chemoreflex response, due to a limited blood flow within and a limited release of metabolites from the muscle. The chemoreflex primarily elevates BP by a sympathetic vasoconstriction (Rowell and O'Leary, 1990), but it also seems to affect sympathetic HR modulation (Iellamo et al., 1999a; Fisher, 2014).

In a previous experiment with matched HR between DYN and ISO, we found ISO to elicit a stronger BP response. At the same time total and vagal modulation indices of HRV were increased, while HRV entropy was reduced (Weippert et al., 2013). Nevertheless, as no isolated muscles groups were active and no respiratory data were measured in this study, there is still a lack of knowledge about the pure influence of contraction mode on circulation, respiration and autonomic control at low HR levels.

Summarizing, whether observations of different autonomic cardiovascular control during DYN and ISO had recourse to the muscular contraction mode itself remains unclear because (a) mostly cardiovascular effects of ISO and DYN had been studied separately, (b) the quantification and thus the application of equivalent workloads during different contraction modes are difficult (Chapman and Elliott, 1988), (c) often not only exercise mode but also the compared muscles size and location was different, (d) most researchers investigated DYN and ISO at higher workloads, probably producing local limitations in blood flow during ISO. Thus, it is not clear whether the different cardiovascular control, if any, is caused by a different metabolic situation during DYN and ISO, changes in central command or a mechanoreceptor feedback due to the different type of contraction.

Therefore, the research question arising from these findings was whether and—if so—how autonomic cardiovascular and respiratory control differed in response to DYN vs. ISO at a similar, low HR level. HRV, the beat to beat fluctuation of HR, and BP were used as non-invasive measures to elucidate the autonomic mechanism underlying cardiovascular control under the different experimental conditions (Task Force of the European Society of Cardiology and the North American Society of Pacing and Electrophysiology, 1996). We hypothesized BP to be higher during ISO if compared to DYN—despite similar HR levels—indicating an increased sympathetic drive to the vessels. At the same time an increased dual autonomic cardiac modulation would be characterized by an increase of total variability assessed by the standard deviation of the R–R series (SDNN). An increased vagal cardiac modulation during ISO should be characterized by an elevated HRV high frequency power (HFP), while increases of sympathetic cardiac outflow should be reflected by an increased normalized low frequency power (LF n.u.). Further, a breakdown of HR complexity has been observed during a coactivation of sympathetic and vagal outflow following cold face immersion (Tulppo et al., 2005). Provided a similar coactivational response pattern during ISO a reduction in HR complexity should be evident. Respiratory measures were used to control for potential contributors of autonomic HR modulation Grossman and Taylor (2007).

## Material and methods

### Ethics statement

This study was performed in compliance with the Declaration of Helsinki and approval of the local ethics committee at the University of Rostock was obtained.

### Participants

Twenty-two healthy males were recruited by personal invitation and gave their informed written consent to take part in this study. Table 1 shows selected characteristics of the participants. All volunteers were physically active and healthy and none of them took medication. They abstained from any exhaustive exercise and alcohol for 24 h prior to the experiment. Further, the consumption of caffeine or nicotine was not allowed during the night and on the morning of the experiment.

**Table 1:** **Characteristics of the participants (*****N*** **= 22)**.

	**Age [yrs]**	**Weight [kg]**	**Height [m]**	**BMI [kg/m^2^]**
Mean	26.7	76.9	1.81	23.4
SD	3.6	7.2	0.06	2.2
Range	21.0–36.0	62.9–87.4	1.71–1.94	19.0–28.5

### Protocol and study design

**Figure 1:**
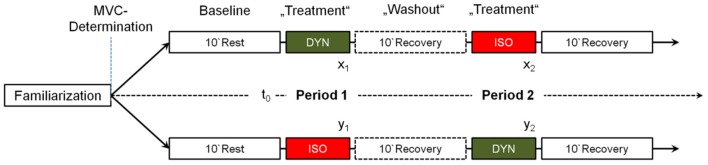
**Design of the experimental cross-over study (*****N*** **= 22)**.

**Figure 2:**
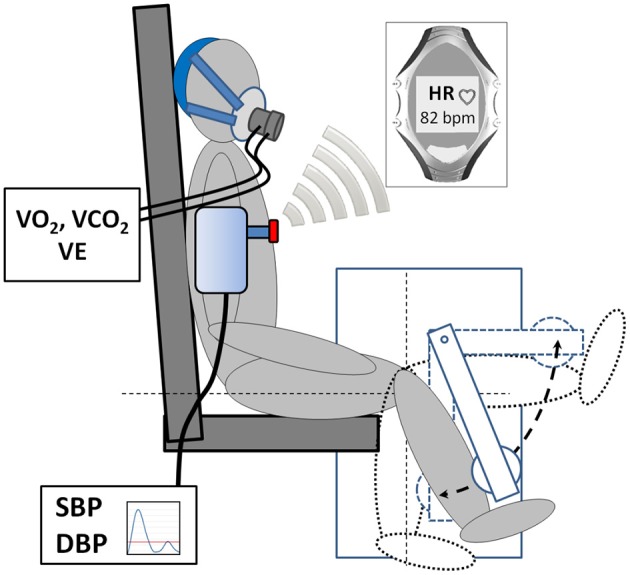
**Scheme of the experimental setup**. Isometric and dynamic exercises were controlled by a CYBEX NORM dynamometer (Computer Sports Medicine® Inc., Stoughton, MA, USA). Heart rate was monitored continuously using a Polar® heart rate monitor (Polar® Inc., Finland). Blood pressure was measured discontinuously during the last minute of each exercise intervention. Respiratory gas analysis was carried out breath-by-breath using the Metamax 3B system (Cortex Biophysics Inc., Germany).

The experimental design is visualized in Figure 1. After familiarization with the experimental setup (Figure 2) isometric maximum voluntary torque of the right quadriceps femoris muscle was determined at 70° of knee flexion (0° = full extension) using a CYBEX NORM dynamometer (Computer Sports Medicine®, Inc., Stoughton, MA, USA). The axis of the dynamometer was aligned with the anatomical knee flexion-extension axis, and the lever arm was attached to the anterior aspect of the shank 2–3 cm above the lateral malleolus. Straps across the waist and the chest prevented excessive movements. Isometric maximum voluntary torque was tested by asking the subjects to exert isometric knee extensions against the lever arm of the dynamometer for 3 s. For each trial, subjects were thoroughly instructed to act as forcefully and as fast as possible. They were motivated by strong verbal encouragement and online visual feedback of the instantaneous dynamometer torque provided on a digital oscilloscope (HM1508, HAMEG Instruments, Germany). A rest period of 1 min was allowed between the trials. The maximal attempts were recorded until the coefficient of variance of three subsequent trials was below 5%. Thereafter, participants were randomly assigned to either sequence groups DYN—ISO or sequence group ISO—DYN (Figure 1). Either contraction mode was performed one-legged with the right quadriceps femoris muscle for 5 min. Prior to the first exercise treatment, a period of 10 min served as physiological baseline. During the whole experiment participants stayed in the same position on the dynamometer. To avoid carry-over effects, a 10 min recovery period served as “washout” period between the two exercise treatments. Contraction intensity of the first exercise treatment (DYN or ISO, respectively) was regulated to reach a significant, but moderately increased HR steady state. Contraction intensity of the second exercise session was regulated to match an equivalent HR level. Torque output during ISO was monitored by an oscilloscope (HM1508, HAMEG Instruments, Germany). Knee flexion angle during ISO was 70°. During DYN, the range of motion was 90° (0° = full extension) and contraction frequency was 0.5 Hz, respectively (Figure 2). Table 2 provides mean values, standard deviations, and ranges for isometric maximum voluntary torques as well as absolute and relative torques applied during ISO and DYN. For real time HR monitoring and the measurement of R–R intervals, a Polar® HR monitor with an accuracy of 1 ms (Weippert et al., 2010) was used. All experimental periods included the measurement of SBP and DBP using the automatic BP measuring device Bosotron 2 (boso Inc., Germany). Mean arterial pressure (MAP) was calculated by (SBP + 2^*^DBP)/3. Further, rate pressure product (RPP) was calculated by HR^*^SBP. Breathing frequency (BF), minute ventilation (VE), tidal volume (V_t_), oxygen uptake (VO_2_), and carbon dioxide output (VCO_2_) as well as the respiratory exchange ratio (RER) and end-tidal oxygen partial pressure of oxygen (PET O_2_) and carbon dioxide (PET CO_2_) were continuously measured breath-by-breath using the mobile metabolic gas analyzer Metamax 3B (Cortex Biophysics Inc., Germany). Further, capillary blood samples were collected from the earlobe within the first minute after the cessation of each experimental session to determine blood lactate concentrations (LAC) (LactateScout, SensLab Inc. Germany). Immediately after each exercise session, participants rated their perceived exertion (RPE) on a Borg-scale from 6 (minimal) to 20 (maximal exhaustion).

**Table 2:** **Isometric maximum voluntary torque (iMVT), absolute and relative torques applied during ISO and DYN (*****N*** **= 22)**.

	**iMVT [Nm]**	**Absolute torque during ISO [Nm]**	**Absolute torque during DYN [Nm]**	**Relative torque during ISO [%iMVT]**	**Relative torque during DYN [%iMVT]**
Mean ± SD	293.1 ± 44.4	34.2 ± 7.7	3.9 ± 3.2	11.8 ± 2.7	1.4 ± 1.3
Range	207.7–384.0	24.0–56.1	0.1–13.0	7.3–20.9	0.03–5.0

### Data processing

To ensure steady state conditions only the last 3 min of each session were analyzed. Data of the respiratory analysis (breath-by-breath) as well as HR (beat-by-beat) were averaged for the 3 min. BP was measured in the last minute of each exercise session. A short term HRV-analysis was performed for 3-min R–R interval segments during steady-state conditions. R–R series were processed using the free software Kubios HRV 2.1 (University of Kuopio, Finland). All analyzed R–R time series exhibited low noise (rate of erroneous R–R intervals below 5%). Before the computation, R–R time series were corrected for artifacts using adaptive filtering. SDNN and frequency measures were calculated. Frequency analyses were performed using a Fast Fourier Transform (Welch's periodogram: 256 s window with 50% overlap). The power in the high frequency range (HFP) is a measure of the vagal cardiac efferent activity, while the power in the low frequency range (LFP) is modulated by both autonomic branches. The normalized LF power (LFP/LFP + HFP, LF n.u.) is usually viewed as an index of modulation of the sympathetic branch of the autonomic nervous system. Further, HR sample entropy (SampEn) was calculated. SampEn is a measure of complexity of the R–R interval series with irregularity/complexity resulting in high and regularity in low values, respectively. Highest values are typical for stochastic data sets. SampEn measures the likelihood that runs of patterns that are close to each other will remain close in the next incremental comparisons (Pincus, 1991). Its calculation relies on counts of *m*-long templates matching within a tolerance *r* that also match at the next point. For SampEn calculation the value of *m* was selected to be *m* = 2, for tolerance *r* a fraction of the standard deviation of the R–R data (*r* = 0.2^*^SDNN) was chosen (Pincus, 1991).

### Statistics

The differences between the different contraction modes (“treatments”) were assessed by means of a *t*-test for independent samples using the intra-individual differences between the outcomes in both periods as raw data (Wellek and Blettner, 2012). The statistical procedure is recommended for cross-over design and includes a statistical test to rule out carryover effect. Effect size for the differences between DYN and ISO were calculated using G^*^Power 3.1.9.2 (Düsseldorf University, Germany).

## Results

The preliminary statistical test proved the assumption that a carryover effect could be ruled out as *p*-values of all measures for the preliminary test were > 0.05.

Average HR raised moderately from 68 ± 8 bpm at baseline to 82 ± 8 bpm during both types of exercise. During the exercises a HR steady state could be established and workload intensity was perfectly matched by HR for each participant. Muscular contraction mode had a large effect on respiratory measures BF and VE, a medium effect on VO_2_, VCO_2_ and PET CO_2_ and no effect on V_t_, RER, PET O_2_, and LAC. Further, SBP and RPP were moderately, DBP and MAP largely affected by the contraction mode (Table 3).

**Table 3:** **Mean ± SD for baseline and exercise conditions of heart rate, blood pressure, heart rate variability, and subjective effort;** ***p*****-values and effect sizes for comparisons between dynamic (DYN) and static exercise (ISO), (*****N*** **= 22)**.

**Parameter**	**Baseline before DYN**	**Baseline before ISO**	**DYN**	**ISO**	***p*-Value**	**Effect size (Cohen's *d*)**
Heart Rate [bpm]	68.3 ± 8.0	68.4 ± 8.0	81.8 ± 7.7	82.1 ± 7.6	0.492	−0.153
Lactate conc. [mmol/L]	1.4 ± 0.3	1.3 ± 0.4	1.3 ± 0.4	1.3 ± 0.3	0.786	−0.060
BF [1/min]	15.2 ± 3.3	15.1 ± 3.2	21.4 ± 2.9	17.4 ± 2.9	0.000	2.089
VE [L/min]	9.8 ± 1.4	9.7 ± 1.4	17.3 ± 2.6	13.8 ± 2.6	0.000	1.281
VO_2_ [L/min]	0.30 ± 0.15	0.28 ± 0.15	0.62 ± 0.31	0.47 ± 0.20	0.002	0.955
VCO_2_ [L/min]	0.26 ± 0.12	0.24 ± 0.12	0.55 ± 0.27	0.43 ± 0.19	0.001	1.034
PET O_2_	108.8 ± 5.0	109.6 ± 6.0	106.9 ± 5.1	108.6 ± 6.7	0.182	−0.340
PET CO_2_	36.9 ± 3.4	36.6 ± 4.5	39.8 ± 3.7	38.4 ± 4.7	0.042	0.559
V_t_ [L]	0.66 ± 0.11	0.66 ± 0.12	0.82 ± 0.13	0.80 ± 0.12	0.523	0.147
RER	0.87 ± 0.18	0.85 ± 0.24	0.89 ± 0.16	0.93 ± 0.20	0.310	−0.253
SBP [mmHg]	126.0 ± 9.3	128.1 ± 8.4	149.2 ± 10.7	156.4 ± 13.1	0.019	−0.545
DBP [mmHg]	79.8 ± 8.1	78.7 ± 7.2	80.1 ± 8.8	93.8 ± 9.2	0.000	−1.391
MAP [mmHg]	95.2 ± 7.2	95.2 ± 6.1	103.1 ± 8.2	114.7 ± 9.3	0.000	−1.199
RPP [mmHg/min]	8644.5 ± 1301.2	8754.6 ± 1151.4	12150.1 ± 1377.1	12787.0 ± 1568.7	0.020	−0.539
R–R interval [ms]	896.7 ± 100.6	894.1 ± 100.8	742.2 ± 70.6	740.3 ± 68.1	0.640	0.105
SDNN [ms]	73.4 ± 28.5	68.1 ± 23.4	38.9 ± 16.8	47.8 ± 20.5	0.003	−0.672
lnHFP [ms^2^]	6.6 ± 0.8	6.5 ± 0.9	5.1 ± 1.2	5.7 ± 0.9	0.002	−0.789
lnLFP [ms^2^]	7.4 ± 1.0	7.3 ± 0.9	5.9 ± 0.9	6.7 ± 0.8	0.000	−0.987
LF n.u.	64.2 ± 16.7	65.8 ± 13.9	66.3 ± 19.7	71.3 ± 13.7	0.087	−0.409
SampEn	1.45 ± 0.30	1.44 ± 0.28	1.60 ± 0.31	1.30 ± 0.23	0.000	1.034
RPE	6.5 ± 0.8	6.5 ± 0.7	12.2 ± 1.3	15.1 ± 1.4	0.000	−1.386

BF, breathing frequency; VE, minute ventilation; VO_2_, oxygen uptake; VCO_2_, carbon dioxide output; PET CO_2_, partial pressure of end tidal CO_2_; PET O_2_, partial pressure of end tidal O_2_; V_t_, tidal volume; RER, respiratory exchange ratio (VCO_2_/VO_2_); SBP, systolic blood pressure; DBP, diastolic blood pressure; MAP, mean arterial pressure; RPP, rate pressure product; R–R interval, heartbeat-to-heartbeat interval; SDNN, standard deviation of the R-R intervals of the record; lnHFP, natural log-transformed high frequency power; lnLFP, natural log-transformed low frequency power; LF n.u., LFP in normalized units; SampEn, sample entropy; RPE, perceived exertion rated on a Borg scale from 6 (= minimal) to 20 (= maximal exhaustion); N = 22.

HRV total variability index SDNN, as well as lnHF- and lnLF-Power were significantly higher during ISO. The elevation of LF n.u. during ISO approached significance. Conversely, HR complexity measure SampEn was significantly lower during ISO. Compared to DYN, RPE was higher during ISO (Table 3).

## Discussion

The aim of this study was to assess circulatory and respiratory responses during ISO and DYN of the lower limb at the same HR level to elucidate the mechanisms of autonomic control during the different contraction modes. Human studies that compared cardiovascular responses to forearm DYN and ISO at equivalent workload quantified by time-tension index (TTI) yielded no differences for HR, SBP, DBP, MAP, RPP, stroke volume and RPE when time was variable and peak tension was hold constant at 30% MVC (Stebbins et al., 2002). However, when time was hold constant and peak tension during DYN increased to 60% to give a similar TTI, all these variables were significantly higher during DYN. Similar results have been found in an animal model (Daniels et al., 2000). Our results seem to be contrary to these results, but it has to be considered that we not only used one-legged compared to handgrip exercise but also a different approach to make DYN and ISO equivalent. We matched intensities in terms of physiological strain, quantified by HR for two reasons: First, using a similar TTI with time being the constant variable would *per se* give different results due to the significantly higher peak tension to be generated during DYN. As has been shown previously, keeping peak tension constant would result in similar physiological responses during DYN and ISO (Daniels et al., 2000; Stebbins et al., 2002). However, due to the extended time spend in DYN, the physiological costs are not similar between TTI matched exercises. Second and more importantly for the scope of the present experiment, matching DYN and ISO by HR helps to answer the question whether the mode of cardiac autonomic control differs between the two types of exercise, despite resulting in the same net effect on HR (Kollai and Koizumi, 1979; Berntson et al., 1993).

Although energy consumption increased during DYN, reflected by an increased oxygen uptake, LAC concentration and RER support the view of a sufficient oxygen supply and thus comparable metabolic situations of the working muscle during both kinds of exercise. Despite these findings and the same net effect of DYN and ISO on HR, autonomic control of cardiovascular and respiratory responses seemed to be different. While respiratory response was lower, BP was higher under ISO. VE under ISO was lower due to the lower respiratory rate (-20% compared to ISO). Further, total R–R variability was higher, while R–R complexity, measured in terms of entropy, was lower during ISO. RPP, an indirect measure of myocardial oxygen consumption, was higher by 5% under ISO due to the elevated SBP, and also RPE was significantly higher.

A similar or even lower stroke volume during low intensity ISO vs. DYN (Lind and Mcnicol, 1967; Helfant et al., 1971; Laird et al., 1979; Hietanen, 1984; Stebbins et al., 2002; Crisafulli et al., 2003; Elstad et al., 2009) gives evidence for the peripheral vasoconstriction being the main modulator of the different BP response observed during ISO and DYN. Especially chemical stimulation by metabolite accumulation and mechanical stimuli as well as influences from higher brain centers (central command) can lead to the enhanced BP and HR response (Abboud, 1979; Rotto and Kaufman, 1988; Rowell and O'Leary, 1990; O'leary, 1993; Iellamo et al., 1999b; Carrington et al., 2001). It is possible that muscle chemo- and/or mechanoreflexes override the baroreflex during ISO (Ponikowski et al., 1997; Hartwich et al., 2011), resulting in an increased sympathetic activation with a subsequent vasoconstriction; either induced by direct sympathetic discharge at the vasculature (Mark et al., 1985) and/or indirectly via the increase of circulating catecholamines (Krzeminski et al., 2002). In our experiment mechanical stimuli might have played the dominant role. Despite the stronger perception of effort during ISO—the mean RPE value corresponded to “hard” or “heavy”—a metabolite accumulation resulting in an increased chemoreceptor feedback from the working muscle is not very likely, because the low intensity of ISO in our experiment should have prevented a compromise in muscle blood flow (Kilbom and Persson, 1982; Sejersted et al., 1984; Sjogaard et al., 1988). As participants were fixed in the identical positions during the exercises, by the use of the featured belts of the Cybex, for keeping the body position during the experiments no extra muscular effort was necessary. Further, participants were instructed not to use additional muscle effort, but the quadriceps femoris. Thus, it seems to be not very likely that additional muscular action has significantly contributed to the HR increase. However, we did not control for this by additional EMG-measurements. An explanation for the apparently high RPE despite the low exercise intensity might be that RPE has been developed and evaluated for dynamic, whole body activity, such as cycling. Thus, subjective evaluation of perceived load might systematically differ between these types of exercise. Further, despite the low exercise intensity, isometric, one-legged work with the applied knee-flexion is an unaccustomed activity of a muscle, predominantly working in a dynamic mode.

The HR steady states during DYN and ISO, identical RER—speaking for low exercise intensity—as well as the similar low LAC concentrations during DYN and ISO also support the view that mechanoreceptor feedback rather than different chemoreceptor stimulation plays the dominant role in the distinct circulatory and respiratory responses. It can be argued that muscle mass during low intensity one-legged exercise is too small to significantly increase LAC concentration assessed at the earlobe. However, it has been shown in previous studies that an increase of blood lactate can principally be determined after one legged exercise, even when blood samples are taken from other sites than the femoral veins (Stamford et al., 1978). Other work also supports the view that mechanoreceptor afferent activity might be a main contributor of the distinct autonomic circulatory and respiratory responses under the different contraction modes. In an experiment, applying sustained passive stretching (comparable with ISO) and rhythmic passive stretching (comparable with DYN) of the right triceps surae muscle, Gladwell and Coote (2002) found distinct cardiovascular responses to these stretching modes. Continuous stretching activated type III and IV afferents and led to significant BP and HR increases, whereas rapid rhythmic stretch—activating group I and II muscle afferents—did not. Results from other studies have also shown that mechanosensitive afferent type III and IV fibers contribute to the cardiovascular response to ISO (Kaufman et al., 1983; Waldrop et al., 1984).

Based on the results the working hypothesis, claiming a different cardiovascular response pattern to DYN and ISO, can be accepted. Beside the BP response that seems to evidence increased sympathetic activation during ISO, significantly higher absolute HRV spectral power in the LF and HF range was found. As hypothesized, the significantly higher HF power indicates an increased vagal HR modulation during ISO. At the same time, there was as statistical trend for a moderate effect of contraction mode on LF n.u. Although modulated by both autonomic branches and the precise physiological mechanism still debated, LF-Power is believed to reflect cardiac sympathetic activity when expressed in normalized units (Pagani et al., 1986; Task Force of the European Society of Cardiology and the North American Society of Pacing and Electrophysiology, 1996). Thus, beside an enhanced vagal HR modulation, the elevated LF n.u. can be cautiously interpreted as an increase in the portion of sympathetic HR modulation. Despite the portion of sympathetic effect on the heart being increased as compared to DYN, the net effect of HR might be blunted due to an absolute increase of vagal efferent activity. In an early animal experiment Kollai and Koizumi (1979) found that “changes in heart rate were correlated well with alterations in vagal activity until the increase in sympathetic activity became greater, then the two curves were not parallel.” A sympatho-vagal coactivation might provide precise control and tuning of the cardiac function (Berntson et al., 1991). Further, the coordination of neural input to the ventricular muscle (inotropic effect of the sympathicus) and sinoatrial node (vagal outflow for HR) can maximize cardiac output (Kollai and Koizumi, 1979; Paton et al., 2006). In animal studies a coactivation had been shown to occur after chemoreceptor activation (Kollai and Koizumi, 1979). Autonomic nervous system coactivation increased rhythmic HR fluctuations in an animal model (Kollai, 1981), which is in line with our finding of increased SDNN. Complementary to traditional HRV-measures, which give information on the magnitude of the variability or important rhythms, non-linear indices are able to identify complex patterns of the analyzed time series. In our experiment the increase of SDNN and HRV spectral power was accompanied by a decrease of SampEn during ISO, speaking for loss of complexity under this exercise condition. However, it is not clear whether these changes originate from sympathetic and/or vagal HR modulations or other mechanisms. Some studies suggest that non-vagal influences can contribute to changes in HR complexity (Tulppo et al., 1996), while others suggest vagal (Porta et al., 2013) and/or sympathetic (Porta et al., 2007b) contributions. During head-up tilting a change from vagal to sympathetic dominance resulted in a significant decrease of SampEn (Porta et al., 2007a). Based on this finding we can cautiously conclude that compared to DYN, sympathetic drive is increased in response to ISO as well. Interestingly, using an animal model Kollai and Koizumi (1979) found that reciprocal action between the two autonomic nerves tended to produce unstable oscillatory heart beats and that cardio-vagal coactivation “stabilizes the heart.” During vagal-sympathetic coactivation at rest, induced by cold face immersion, Tulppo et al. (2005) found a breakdown of HR complexity in human subjects. These findings are in line with our findings of higher regularity/lower complexity during ISO. An autonomic coactivation under ISO might lead to a reduction of entropy by a stabilizing effect on HR. Summarizing, results of this experiment confirm findings from our previous study (Weippert et al., 2013). The results evidence a significant impact of exercise mode on cardiovascular control and support our hypothesis of an increased dual HR modulation during ISO. Results from the HRV analyses speak clearly for a stronger vagal heart rate HR modulation during ISO if compared to DYN. The findings of similar HRs, a trend for an increased LF n.u. and a reduced HR complexity lead to the assumption that also sympathetic mechanisms are involved and further support the assumption of an autonomic coactivation under ISO. However, a limitation of our experiment is the lack of continuous BP measurements, which might have given additional insight into SBP variabilities and baroreflex sensitivity and thus underlying mechanisms.

Analysis of respiratory gas analysis confirm a negligible influence of respiration on HRV spectral bands under the experimental conditions, because average breathing frequency of all participants was well within the HF range during both exercise modes and the difference between DYN and ISO was 4 cycles/minute only. From these results it can be assumed that (1) there is no confounding effect due to a shift of the respiration related HRV spectral band into the LF range, (2) the effect on baroreflex sensitivity due to the decreased breathing rate should be—if at all—small (Bernardi et al., 2002) and (3) the finding of *V*_t_ being similar during both exercises indirectly supports the assumption of similar pulmonary stretch receptor afferent activity during both exercises.

Concluding, the results of this experiment evidence distinctive patterns of physiological response for DYN and ISO during one-legged exercise. Despite the same HR response, subjective, respiratory, and circulatory responses differed significantly between one-legged ISO and DYN. Compared to DYN at identical low HRs, the physiological workload responses to sustained ISO are characterized by higher BP, lower breathing rate, larger HRV spectral power in the low and high frequency range and different HR complexity. Results of our study support the assumption that under low intensity exercise mechanoreceptor feedback from the working muscle rather than chemo- or baroreceptor afferent activity plays a substantial role in the distinct autonomic circulatory and respiratory responses to DYN and ISO. The change of mechanoreceptor feedback might induce a stronger sympathetic efferent activity to the vessels, leading to the BP increase during low intensity ISO. Further, compared to DYN contractility of the heart might be slightly higher during ISO to counteract the increased arterial pressure. This indirectly points to an increased sympathetic outflow under ISO, associated with increased vagal modulation to result in a similar HR as during DYN. Further, an interaction of mechanoreceptor feedback and influences from higher brain centers (central command) on cardiovascular control might contribute to the distinct cardiovascular response as well. Principally, a change of central command, mirrored by the increase in subjective effort, can elevate BP (Goodwin et al., 1972; Rowell and O'Leary, 1990; Iellamo et al., 1997b; Carrington et al., 2001; Iellamo, 2001; Williamson et al., 2006; Smirmaul, 2012) and HR (Rowell and O'Leary, 1990) and thus have to be considered as a possible contributor of the different autonomic cardiac activity. Recent work has shown that central command—in contrast to the traditional view—rather increases sympathetic efferent activity than decreasing vagal nerve traffic during exercise (Matsukawa, 2012). From the results it can be concluded that, compared to DYN, ISO seems to increase vagal and sympathetic cardiovascular outflow. The concomitant increase of vagal cardiac drive during ISO opposes the sympathetic effect on HR leading to the same net effect on HR during both contraction modes. Further, a coactivation might effectively control cardiac output during ISO. However, as direct measurement of sympathetic or vagal outflow to the heart is not practical in humans assumptions regarding a cardiac sympatho-vagal coactivation during ISO has to be drawn cautiously. Despite HRV indices support this interpretation, future research should use multivariate strategies, involving additional signals. Whether, mechanoreceptor feedback alone, a change in central command, or the interaction of both mechanisms is the main contributor of the distinct autonomic responses to the different exercise modes remains to be elucidated.

## Author contributions

MW, MB, and KB designed this study; MW, MB, RG collected the data; MW, MB, RG, and KB analyzed the data, MW interpreted the data; MW and MB drafted the manuscript, all authors revised the manuscript and approved the final version to be published.

### Conflict of interest statement

The authors declare that the research was conducted in the absence of any commercial or financial relationships that could be construed as a potential conflict of interest.
